# Community voices to understand and promote liveability in the Green Corridor urban transformation project in Bogotá, Colombia

**DOI:** 10.1186/s12889-026-27578-9

**Published:** 2026-05-16

**Authors:** Lina María Gómez-García, Elena Mª Gras-García, Eduardo De la Vega-Taboada, Nicolás Solórzano Duran, Abby C. King, Zakaria Nadeem Doueiri, Jose Mario Mayorga, Carlos M. Moncada, Luis A. Guzman, Laura Mejía Riveros, Olga L. Sarmiento

**Affiliations:** 1 School of Medicine, Universidad de los Andes, Carrera 1 N 18ª-12, Bogotá, Colombia; 2 Department of Epidemiology & Population Health, Stanford University School of Medicine, Stanford, CA USA; 3 Our Voice Global Citizen Science Research Initiative, Stanford, CA USA; 4 College of Nursing, Center of Population Science for Health Empowerment, Florida State University, Tallahassee, FL USA; 5 Department of Medicine (Stanford Prevention Research Center), Stanford, CA USA; 6 Department of Civil and Agricultural Engineering, School of Engineering, Universidad Nacional de Colombia, Bogotá, Colombia; 7 Department of Civil and Environmental Engineering, Grupo de Sostenibilidad Urbana y Regional SUR, Universidad de los Andes, Bogotá, Colombia

**Keywords:** Citizen science, Community-based participatory research, Our Voice, Liveability, Climate change mitigation, Climate change adaptation, Urban planning, Latin America

## Abstract

**Background:**

Latin American cities are transforming in response to climate change, yet residents’ liveability experiences before these transformations begin are rarely documented. In Bogotá, the 2022–2035 Land Use Master Plan’s Green Corridors aim to promote sustainable mobility and integrate green infrastructure in urban design, with the first corridor planned along 7th Street. This study provides a baseline evaluation of liveability conditions along 7th Street before construction, aiming to (i) identify perceptions of social and built environment factors that facilitate or hinder liveability and (ii) document potential solutions to identified barriers.

**Methods:**

We employed a multi-method approach. A cross-sectional household survey described residents’ sociodemographic profile and perceptions of the Green Corridor’s expected effects on liveability. The *Our Voice* citizen science method engaged residents and commuters in identifying perceived facilitators and barriers, proposing potential solutions, and exchanging knowledge with policymakers.

**Results:**

Residents’ most expected improvements included increased vegetation, pedestrian and cycling infrastructure, and public space upgrades, while the most anticipated deteriorations included difficulty in car use and worsening public transport operations. Through the *Our Voice* method, 66 citizen scientists captured 1123 photo-narratives, documenting access to essential services, green areas, and urban trees as the most mentioned facilitators, while poor pedestrian infrastructure and safety concerns as the most mentioned barriers. Proposed solutions included infrastructure improvements, educational campaigns, and evidence-based policymaking. The process increased environmental awareness among citizen scientists and supported knowledge exchange with policymakers.

**Conclusion:**

This study underscores the importance of grounding climate mitigation and adaptation efforts in baseline assessments of urban residents’ liveability experiences. Built environment factors were widely valued as facilitators, while social environment factors emerged as persistent barriers, a pattern that the *Our Voice* method deepened by revealing the conditions that underpin these perceptions. The participatory process generated practical solutions, deepened citizen scientists’ awareness of their surroundings, and enabled meaningful engagement with policymakers. As cities pursue climate goals, these findings offer a foundation for understanding and evaluating the effects of a Green Corridor on residents’ everyday liveability as the intervention unfolds.

**Supplementary Information:**

The online version contains supplementary material available at 10.1186/s12889-026-27578-9.

## Background

Latin America is experiencing an increase in the frequency of extreme weather events, which threaten people’s livelihoods and health [1–4]. Urban areas are particularly vulnerable to climate-related hazards, which are further exacerbated by underlying social challenges, including resource inequality, poverty, informal housing, and high population density [5, 6]. City planning and design can address the challenges of climate change, public health, and social inequality. By shaping the built environment, planning and design strategies can reduce climate-related risks while fostering healthier, more equitable communities [7–10].

**Fig. 1:**
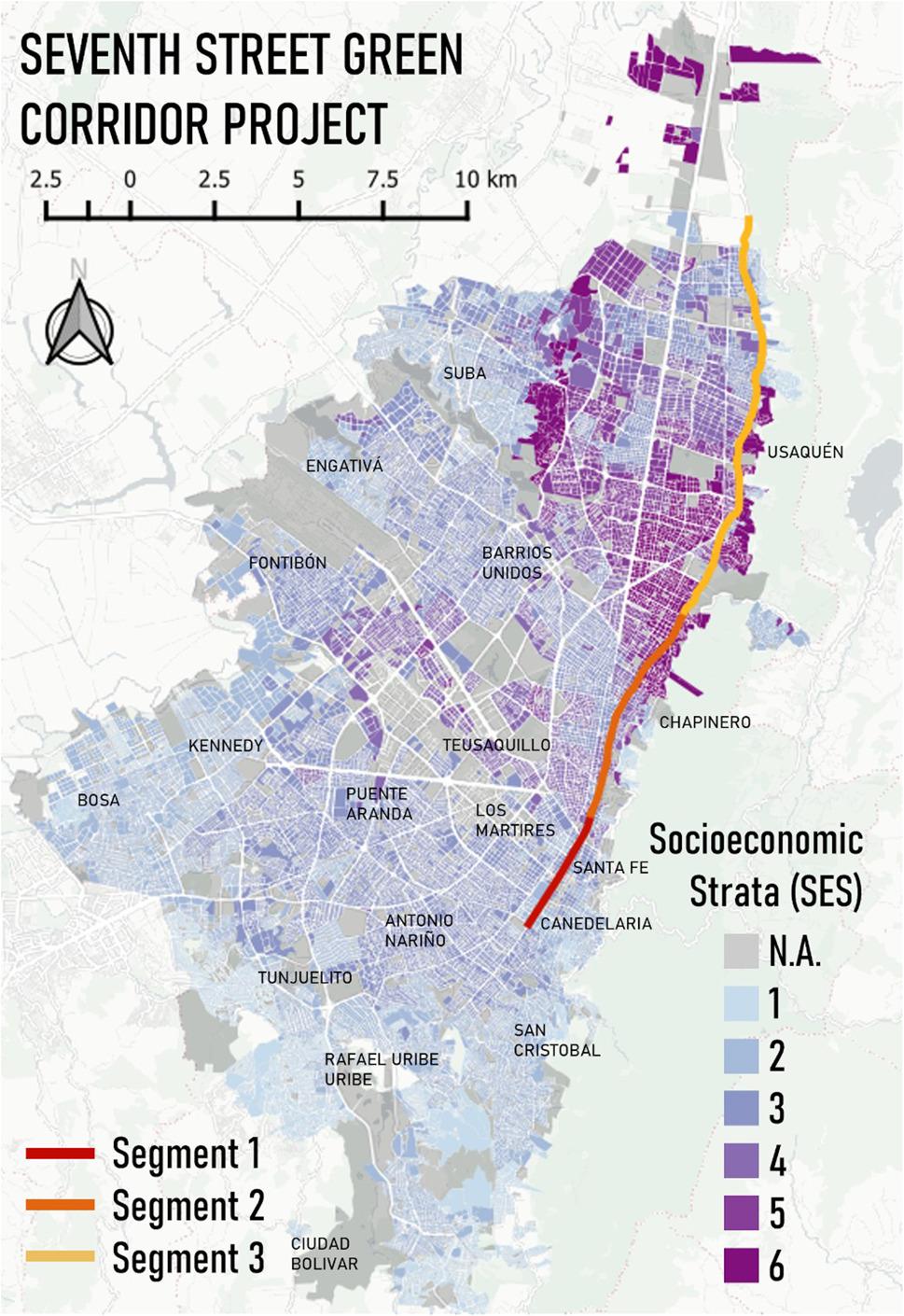
Map of 7th Street and projected segments of the Green Corridor urban transformation. The map depicts 7th Street location in Bogotá, overlaid with the city’s official socioeconomic strata based on cadastral property data, highlighting how the street intersects areas of varying income levels. Each color-coded segment of 7th Street represents a segment of the Green Corridor urban transformation

Across Latin America, cities are adopting a variety of strategies to mitigate and adapt to climate change [2, 11, 12]. In Colombia, these efforts are structured through Land Use Master Plans, which set the overarching framework through which cities respond to long-term social, environmental, and economic challenges [13]. In 2022, Bogotá revised its Land Use Master Plan to strengthen its response to climate change while improving residents’ quality of life [14]. Among the strategies adopted in the new plan is the development of a network of Green Corridors, designed to promote sustainable mobility by reallocating street space to prioritize public transit, cycling, and walking [14]. They also incorporate green infrastructure, including expanded tree canopies, vertical gardens, parks, plazas, and urban drainage systems, to enhance climate resilience and improve urban environmental quality [14]. The first of 32 Green Corridors is planned for 7th Street, a 24 km arterial road connecting the city center with the north. Its design divides 7th Street into three geographic segments based on the unique social and built environment characteristics of the area (Fig. 1). Construction is scheduled to begin in 2026 and be completed in 2030 [15].

**Fig. 2:**
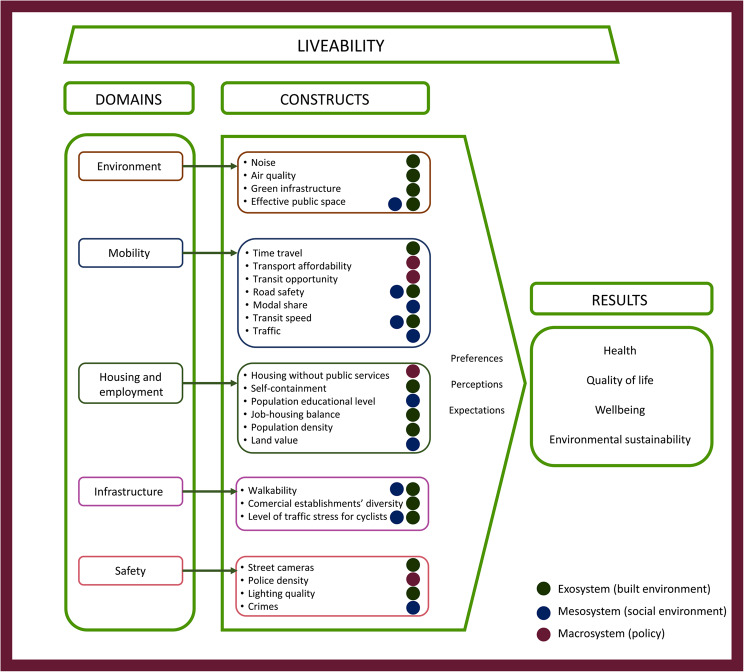
Liveability framework designed for the city of Bogotá. The figure depicts the five domains of urban liveability and illustrates how each domain is operationalized through specific constructs across the exosystem (built environment), mesosystem (social environment), and macrosystem (policy)

In this context, we use the liveability framework designed for the city of Bogotá to examine the conditions residents perceive as shaping their health and wellbeing in the Green Corridor area [16]. Liveability is a multidimensional construct arising from the interrelationship among variables across five domains within the mesosystem (social environment) and exosystem (built environment) (Fig. 2), all of which can be modified by decisions at the macrosystem (policy) [17, 18]. The five domains that capture the conditions underpinning urban life in Bogotá are the (i) environment, which reflects the quality of ecological conditions of a city, (ii) mobility, which relates to the availability, safety, and accessibility of transport options and the conditions of movement through the city, (iii) infrastructure, which encompasses the availability and accessibility to social and urban services that support daily functioning, (iv) housing and employment, which capture residential conditions, service provision, and access to education and employment opportunities, and (v) safety, which reflects both objective and perceived conditions that enable people to occupy and use urban space. These variables interact across scales and population groups, shaping differentiated experiences of health, wellbeing, and quality of life within a city.

Understanding liveability requires qualitative accounts that capture residents’ experience of urban environments. Qualitative narratives provide complementary insights into the ways urban spaces shape experiences, perceptions, preferences, and expectations, which in turn influence health, wellbeing, and overall quality of life [19]. *Our Voice* is a citizen science method that enables citizens to be involved in the data collection, analysis, and diffusion to identify relevant factors of the built environment and provides a way to advocate for change [20].

Given that the Green Corridor affects key dimensions of liveability, examining the everyday experiences and expectations of those who inhabit the area is essential for establishing a baseline from which future changes in liveability can be understood. This study, conducted as part of the CIVICA (Liveable and Connected Smart City in Spanish) and SALURBAL-Climate (Urban Health in Latin America) projects, aimed to (i) identify residents’ and commuters’ perceptions of social and built environment factors that facilitate or hinder liveability and (ii) document potential solutions to the identified barriers.

## Methods

### Study setting

Bogotá, the capital of Colombia, is home to approximately 7.9 million inhabitants [21]. Approximately 268,000 residents live, and 347,000 individuals work within an 800-meter buffer zone surrounding 7th Street [22]. Mobility data indicate that 39.1% of trips are made using buses and the Bus Rapid Transit system, while 24.7% are completed on foot and 3.8% by bicycle [23]. More details are available online [24].

### Study design

We conducted a multi-method study combining quantitative and qualitative methods. The quantitative component involved a cross-sectional household survey representative of the adult population living along the Green Corridor to document residents’ sociodemographic profiles and their perceptions of the liveability factors expected to improve or deteriorate with the Green Corridor, providing a population-level baseline before the intervention. The qualitative component employed the *Our Voice* citizen science method with a group of residents and commuters to identify perceived facilitators and barriers to liveability within the social and built environments of 7th Street and propose actionable solutions. While the survey captures the population-level context necessary to situate and interpret the qualitative findings, the insights from participatory citizen science reveal what those conditions mean in everyday urban life. The study was reviewed and approved by the Institutional Review Board of the Universidad de Los Andes in Bogotá, Colombia (Minute No. 2023053011 and Minute No. 2023080118).

### Quantitative component: household survey

Between September 2023 and May 2024, we conducted a cross-sectional household survey representative of the adult population living along each projected segment of the Green Corridor. The survey assessed residents’ perceptions of Green Corridor’s anticipated effects on liveability through a combination of question formats: a 5-point Likert scale measuring agreement with the proposed transformation, and open-ended questions about the three most expected improvements and deteriorations because of the intervention. An English-language version of the questionnaire is provided in Additional File 1.

#### Household survey’s sample

The survey population consisted of adults aged 18 and over who had lived within an 800-meter radius of 7th Street for at least two years and did not plan to move in the next two years. The sample size was determined to achieve a 95% confidence level, with a margin of error of 2,1%. After accounting for a 20% nonresponse rate, the final sample was of 2,445 individuals. The survey followed a stratified sampling approach, based on cartographic block location and inclusion in Bogotá’s 2021 Multipurpose Survey [25]. A subset of the sample was obtained employing a probabilistic, multi-stage design, while another subset followed a non-probabilistic design due to challenges in reaching the intended sample size. In the probabilistic subset, sampling involved the random selection of cartographic blocks, dwellings, households, and one eligible individual per household. In the non-probabilistic subset, cartographic blocks were randomly selected, and individuals were surveyed directly in the street within each selected block. Weighting and non-response adjustments followed the methodology of Valliant, Dever, and Kreuter [26]. A double-robust data integration model was employed to correct potential biases from intercept sampling [27].

#### Household survey’s data analysis

During data collection, interviewers systematically coded open-ended responses into predefined thematic codes, developed to reflect the objectives of the Green Corridor. These codes were deductively categorized using the liveability conceptual framework. Following this transformation, descriptive statistics (percentages) were calculated for all survey items using R Studio. The *survey* package was used to account for the complex survey design and survey weights.

### Qualitative component: *Our Voice* citizen science method

Between August 2023 and March 2025, we collaborated with urban residents and commuters through the four-step *Our Voice* method [28, 29]. Developed by Stanford University, this method combines participatory citizen science and photovoice to support communities in collecting, analyzing, and disseminating their own data [30, 31]. It has been contextually adapted and used in multiple research projects in Colombia [32–35].

#### Our Voice method’s participants’ recruitment

A total of 66 students, employees, retirees, and full-time unpaid caregivers (family members caring for children, people with disabilities, and/or the elderly) were recruited as citizen scientists along 7th Street, seeking balanced representation along the Green Corridor. Eligibility criteria included commuting at least once per week on 7th Street, living or working within an 800-meter buffer of the corridor, and residing in a low-income neighborhood to amplify underrepresented voices. Recruitment used purposive sampling in parks, plazas, and bus stations, complemented by telephone outreach to local grassroots organizations. Informed consent was obtained prior to participation. Citizen scientists received a food voucher valued at $6 USD (COP 25,000) at the end of each meeting.

#### Our Voice method’s four-step procedure

##### Discover

Between August and October 2023, citizen scientists commuted between their homes and main activity locations using their preferred mode of transport (walking, public transport, bicycle, private car) and documented facilitators and barriers to liveability along 7th Street. Using the multi-lingual *Our Voice* Discovery Tool mobile app [36], citizen scientists captured geocoded photos, recorded audio narratives, and rated each photo-narrative as a facilitator, barrier, or both. They responded to the guiding question: “A liveable city is one where neighborhoods are safe, have clean environments, are walkable and bike-friendly, and homes are close to public transport, health, education, cultural, and recreational services. What aspects of your daily life around 7th Street enhance or hinder liveability?” The prompt was previously validated with five residents not involved in the study.

##### Discuss

Between August and October 2023, three 2-hour community meetings were held to organize the collected data (August 26, 2023, September 9, 2023, September 30, 2023). Citizen scientists worked in small groups reviewing their printed photos and verbatim transcripts, grouping them into relevant themes, and organizing them as facilitators or barriers on a display board. Each group then reached consensus on the three most important barriers and brainstormed potential solutions.

##### Activate

On April 4, 2024, citizen scientists and city-level policymakers participated in a 2.5-hour community meeting where findings from the Discovery Tool were presented. Citizen scientists discussed key barriers and proposed solutions, while policymakers addressed concerns and engaged in dialogue about potential actions.

##### Change

Between April 2024 and May 2025, the research team implemented an advocacy strategy to inform the ongoing dialogue on 7th Street transformation. This strategy included a policy engagement component, with in-person and virtual meetings with decision-makers to present findings and advocate for changes in the Green Corridor, and a public communication component, aimed at increasing awareness through multimedia content (digital infographic, podcasts, and Instagram reels). These materials were co-produced with Universidad de los Andes Center of Journalism Studies and the digital media outlet 070. Content highlighted liveability determinants through academic analysis and lived experiences of a public transport user, a cyclist, a person with disabilities, and a senior adult. All materials are available online [37].

#### Our Voice method’s data analysis

The data included: (i) verbatim transcripts from the photo-narratives collected by citizen scientists during the *Discover step* and (ii) transcripts detailing the solutions proposed by citizen scientists in the *Discuss step* and policymakers’ responses to those solutions in the *Activate step*.

##### Discover step photo-narratives

Photo-narratives were analyzed using codebook thematic analysis [38]. During the *Discuss* meetings, citizen scientists initially identified recurring themes. Three researchers with expertise in Social Sciences (LMGG, NSD) and Public Health (CCC) developed a preliminary codebook based on these themes and incorporated additional themes after reviewing all narratives. The finalized codebook was organized employing the liveability framework, grouping themes first into the mesosystem (social environment), exosystem (built environment), and macrosystem (policy), and then within the five domains of environment, housing and employment, infrastructure, mobility, and safety (Fig. 2). Themes were also classified as facilitators, barriers, or both according to participants’ interpretations. Afterwards, five researchers with backgrounds in Social Sciences (LMGG, NSD, MO) and Public Health (CCC, JKBC) independently coded the narratives, assigning up to two themes per narrative. The research team met five times to compare coding decisions and resolve discrepancies.

##### Discuss and activate step transcripts

Transcripts documenting proposed solutions and policymakers’ responses were analyzed using thematic analysis. Two researchers with backgrounds in Social Sciences (LMGG, NSD) independently reviewed the transcripts and categorized each solution and response according to the socioecological level targeted. Disagreements were resolved through discussion across three meetings.

## Results

### Quantitative component: household survey

#### 7th Street residents’ demographic profile

According to the representative household survey, approximately 268,000 people live within an 800-meter buffer surrounding 7th Street (Table 1). The gender distribution is balanced, with 49.3% identifying as women and 50.5% as men. With respect to occupational status, 57.6% of them were employed or working freelance, 11.7% were unpaid caregivers, 6.0% were retired, 5.8% were studying, and 4.3% were unemployed. In terms of educational attainment, 12.8% had completed primary school, 32.3% high school, 33.0% university or technical studies, and 18.0% a graduate program. Household income levels varied: 24.7% reported monthly incomes below $261 USD (COP 1,160,000), while 25.7% reported incomes above $1,044 USD (COP 4,640.000).

**Table 1:** 7th Street residents’ demographic profiles and perceptions on whether the Green Corridor will enhance or hinder liveability

Variable	Overall (*n* = 2,445) (*N* = 268,821)
	%
Demographic profiles	
Sex	
Women	49.3%
Men	50.5%
Occupation	
Working	57.6%
Unpaid caregiving	11.7%
Retired	6.0%
Studying	5.8%
Unemployed	4.3%
Other	6.7%
Last completed level of education	
Preschool or lower	3.8%
Primary school	12.8%
High school	32.3%
University/Technical program	33.0%
Graduate program	18.0%
Monthly household income	
≤ 1 minimum wage (261 USD)	24.7%
> 1 to ≤ 2 minimum wages (522 USD)	22.5%
> 2 to ≤ 4 minimum wages (1,044 USD)	21.2%
> 4 minimum wages	25.7%
Do not wish to respond	5.9%
Perceptions of the Green Corridor project	
Level of agreement with Green Corridor	
Agree	40.8%
Disagree	23.5%
Indifferent	22.1%
Do not wish to respond	13.6%
Positive aspects of the project	
* Liveability environment domain – exosystem (built environment)*	
Air pollution reduction	11.3%
Increased vegetation	23.3%
* Liveability infrastructure domain – exosystem (built environment)*	
Cultural & community gathering spaces	2.1%
Improved pedestrian & cycling infrastructure	17.2%
Infrastructure upgrades	3.1%
Physical activity spaces	4.5%
Public space upgrades	17.2%
* Liveability mobility domain – exosystem (built environment)*	
Improved public transport	11.6%
Road safety enhancement	4.9%
Sustainable mobility	7.5%
* Liveability housing & employment domain – macrosystem (policy)*	
Support for local commerce	1.4%
* Liveability safety domain – mesosystem (social environment)*	
Crime reduction	6.0%
Negative aspects of the project	
* Liveability environment domain – exosystem (built environment)*	
Increased air pollution	5.5%
Vegetation degradation	6.3%
* Liveability infrastructure domain – exosystem (built environment)*	
Limited access to parking lots	5.4%
Pedestrian & cycling infrastructure damage	7.1%
Public space deterioration	8.6%
* Liveability mobility domain – macrosystem (policy)*	
Public transport decline	11.2%
Reduced car accessibility	19.6%
TransMilenio prioritization	11.7%
* Liveability housing & employment domain – macrosystem (policy)*	
Decline in local commerce	4.3%
* Liveability safety domain – mesosystem (social environment)*	
Rising crime rates	9.0%

Table 1 presents the demographic profiles of 7^th^ Street residents and their perceptions regarding the Green Corridor project. Percentages are calculated based on the estimated total population (N) of each segment, not the surveyed sample size (n), to allow for population-level interpretation

#### 7th Street residents’ perceptions of the Green Corridor project

According to the representative household survey, 40.8% of 7th Street residents agreed with the Green Corridor transformation, 23.5% disagreed, and 22.1% were indifferent. The most frequently expected improvements included increased vegetation, pedestrian and cycling infrastructure, and public space upgrades, corresponding to the exosystem (built environment) within the environment or infrastructure domains. Conversely, the most expected deteriorations included increased difficulty with car use, prioritizing the Bus Rapid Transit system over other transport modes, and a decline in public transport services, corresponding to the mesosystem (social environment) and the macrosystem (policy) within the mobility domain.

### Qualitative component: *Our Voice* citizen science method

#### Citizen scientists’ sociodemographics

A total of 66 citizen scientists participated in the research process (35 women and 31 men), with a median age of 49 years (IQR = 27–63). Among them, 57.6% (*n* = 38) were employed or freelance workers, 18.2% (*n* = 12) were students, 13.6% (*n* = 9) were unpaid caregivers, 7.6% (*n* = 5) were retired, and 1.5% (*n* = 1) were unemployed. Regarding education, 7.6% (*n* = 5) had completed primary school, 50.0% (*n* = 33) high school, 34.8% (*n* = 23) university or technical studies, and 6.1% (*n* = 4) a graduate program. Additionally, 37.9% (*n* = 25) identified as community leaders, and 7.6% (*n* = 5) reported having a disability (i.e., visual and mobility-related) (Table 2).

**Table 2:** *Our Voice* citizen scientists’ demographics and *Discover step* community audit characteristics

Variable	Overall
	*n* (%)
Citizen scientists	
Total	66
Gender	
Women	35 (53.0%)
Men	31 (47.0%)
Age: median [Interquartile range – IQR]	49 [27–63]
Occupation	
Working	39 (59.1%)
Studying	12 (18.2%)
Unpaid caregiving	9 (13.6%)
Retired	5 (7.6%)
Unemployed	1 (1.5%)
Last completed level of education	
Primary school	5 (7.6%)
High school	33 (50.0%)
University/Technician	24 (36.4%)
Graduate program	4 (6.0%)
Leadership position in the community	
Yes	25 (37.9%)
No	41 (62.1%)
Had a disability	
Yes	5 (7.6%)
No	61 (92.4%)
Transport mode used in the community audits	
Foot	41 (62.1%)
Public transport	16 (24.2%)
Bicycle	6 (9.1%)
Private car	3 (4.6%)
Photo-audio narratives	
Total	1,123
Facilitators	334
Barriers	702
Facilitators/Barriers	87

Table 2 summarizes the demographic characteristics of citizen scientists along 7^th^ Street. Values are presented as the number of individuals (N) and the overall percentage (%). Median age is reported with the interquartile range (IQR) in brackets

#### Discover: facilitators and barriers to liveability along 7th street

Citizen scientists conducted community audits along 7th Street. Most sessions were carried out on foot (*n* = 41, 62.1%), followed by public transport (*n* = 16, 24.2%), bicycle (*n* = 6, 9.1%), and private car (*n* = 3, 4.6%) (Table 2). A total of 1123 photo and audio narratives were captured. Of these, 334 (29.7%) were classified as facilitators of liveability, 702 (62.5%) as barriers, and 87 (7.8%) as both. The following results correspond to the two most mentioned facilitators and barriers by citizen scientists (Table 3). Full coding results are available in Additional File 2.

**Table 3:**
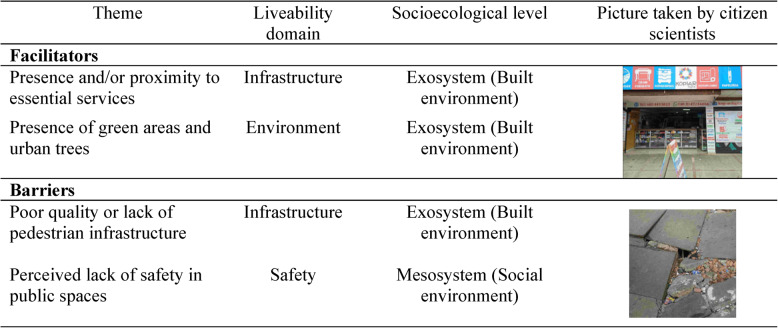
Discover step citizen scientists’ most mentioned liveability facilitators and barriers

Table 3 presents the two most frequently mentioned facilitators and barriers to liveability identified by citizen scientists during the Discover step. Each theme is categorized by the domain (i.e., environment, infrastructure, mobility, housing and employment, safety), and a socioecological level (i.e., mesosystem (social environment), exosystem (built environment), macrosystem (policy)) of the liveability framework for the city of Bogotá

##### Facilitators to liveability

The most mentioned facilitator to liveability was an exosystem (built environment) factor: the presence and/or proximity to essential services, such as hospitals, pharmacies, schools, universities, supermarkets, retail establishments, restaurants, ice cream shops, bakeries, coffee shops, and spaces for physical activity. Citizen scientists noted that access to these services improves their quality of life by fostering social interaction and freeing commuting time for activities meaningful to them.

As one citizen scientist noted:

Regarding access to services such as pharmacies and banks, I believe that there are plenty in my area. There are supermarkets, even if they are small franchise stores (…). There are also many pharmacies, bookstores, and banks (…). (Female citizen scientist, 27 years old).

The second most mentioned facilitator was an exosystem (built environment) factor: the presence of green areas and urban trees, which were praised for improving air quality, reducing respiratory disease, enhancing mental health, and increasing wellbeing. One citizen scientist remarked:

In this segment, breathing already feels a bit easier. The air is cleaner here because the mountains are just 40–50 m away, you’re basically in the hills. 7th Street has a fair number of green areas and trees that help purify the air to some extent, but vehicle pollution remains very strong. (Male citizen scientist, 23 years old).

##### Barriers to liveability

The most frequently mentioned barrier was an exosystem (built environment) factor: the poor quality or lack of pedestrian infrastructure. This includes uneven sidewalks, potholes, obstacles, and a lack of ramps and traffic signals, all of which pose risks for people with disabilities, older adults, and children. According to one citizen scientist:

The sidewalks for pedestrians are a disaster, they’re full of obstacles and poorly designed. 7th Street is terrible for pedestrian accessibility, especially for those who use wheelchairs or face other mobility challenges. (Male citizen scientist, 54 years old).

The second most mentioned barrier was a perceived lack of safety in public spaces due to the mesosystem (social environment). Both men and women citizen scientists attributed this perception to factors such as overcrowded or low pedestrian traffic, the sale and use of psychoactive substances, and the presence of homeless individuals and informal vendors. Only female citizen scientists mentioned street and public transport harassment as a significant concern, particularly in areas with low pedestrian traffic, revealing a gender difference in the experience of safety. As one citizen scientist described:

Men clearly perpetrate sexual harassment against women along 7th Street. When you’re just walking, especially alone, the harassment becomes much more noticeable. People make comments, whistle at you, and sometimes even follow or approach you in invasive ways that can escalate into physical contact, like grabbing your hand or touching you. This happens frequently along this road. This should be addressed, whether through increased police presence, physical emergency help points, or immediate assistance services. This is especially important during late hours when one feels more vulnerable and defenseless. (Female citizen scientist, 21 years old).

#### Discuss: solutions to liveability barriers along 7th Street

A total of 35 citizen scientists attended the three *Discuss step* community meetings. The results below present the solutions they proposed for the themes they prioritized (Table 4).

**Table 4:** *Discuss step* proposed solutions by citizen scientists and *Activate step* policymakers’ responses

Prioritized barriers	Citizen scientists’ proposed solutions	Citizen scientists’ role in policymakers’ strategies	Policymakers’ strategies
Poor quality or lack of pedestrian infrastructure	• Data-driven focalization of areas needing maintenance^*p, b^. • Co-design of pedestrian infrastructure with people with disabilities^*p, b^. • Public space redesign to accommodate people with disabilities^*b^. • Construction of bicycle lanes^*b^.	Deliver *Our Voice* findings to guide priority areas needing intervention	District Secretariat for Mobility centers to report pedestrian infrastructure issues: Local Mobility Centers^*s^.
		No mentioned role – broader policy frameworks and service delivery mechanisms to address concerns	Sustainable Mobility Plan 2023–2035 & Disability Policy 2023–2034^*p^.
Perceived lack of safety in public spaces, including women’s perceptions	• Surveillance cameras^*b^. • Street lighting^*b^. • Police presence^*p^. • Creation of intersectoral civic committees^*p^. • Revitalization of abandoned lots^*b^. • Outdoor gyms^*b^. • Bike-sharing system^*b^. • Co-creation of guidelines on public space use^*p, s^.	Co-responsibility in the design of strategies to transform behaviors	District Secretariats of Safety, Mobility, and TransMilenio education campaigns in public spaces and public institutions to address gender-based violence^*s, p^.
Inappropriate use of public space	• Construction and improved accessibility of public restrooms^*b^. • Educational campaigns on responsible public space use^*s^. • Collaboration to achieve consensus on space governance^*s, p^.	Deliver *Our Voice* findings to guide priority areas needing intervention	District Secretariat for Culture tactical urbanism and education strategy: El Centro Vive^*s, p^.
Waste in public spaces	• Fines^*s^. • Pet ownership taxes^*s^. • Neighborhood awards^*s^. • Educational campaigns^*s^. • Redesign or co-design of waste disposal furniture^*b^. • Improvements to façade aesthetics^*b^. • Tactical urbanism^*b^. • Incentives^*s^.	-	-
Insufficient institutional response to housing barriers	• Subsidies for historical interest housing^*s^.	Co-responsibility in participating on participatory budgets	District Secretariat for Planning Local Planning Units^*p^.
Noise	• Council to monitor compliance with environmental regulations ^*p^. • Education campaigns^*s^. • Urban areas for nightclub establishments^*b, p^.	No mentioned role – broader policy frameworks and service delivery mechanisms to address concerns	District Secretariat for Environment noise control operations^*p^.
Deficiencies in public transport operations	• Addition of new bus routes^*p^. • Addition of new Bus Rapid Transit routes^*p^.	Co-responsibility in reporting issues to improve service operation and monitor fare evasion	TransMilenio mobile app for real-time bus tracking and reporting.
Traffic congestion	• Adjustments to the *Pico y Placa* policy^*p^. • Construction of metro^*b^. • Construction of bicycle lanes^*b^. • Construction of motorcycle lanes^*b^.	-	-
Insufficient institutional response to vulnerable populations’ rights	• Construction of housing complexes in abandoned lots^*b^.	Co-responsibility for the urban space	District Secretariat for Social Integration’s strategy to provide shelter, employment, hygiene products, and civic education to unsheltered individuals^*s, p^.

Table 4 summarizes barriers prioritized by citizen scientists, their proposed solutions, the corresponding strategies implemented by policymakers, and the role of citizen scientists in those strategies. An asterisk (*) indicates the level of intervention addressed by the proposal: s = Mesosystem (social environment), b = Exosystem (built environment), p = Macrosystem (policy). A dash (–) indicates no strategy or role was identified

Citizen scientists proposed several solutions to address the poor quality or lack of pedestrian infrastructure. These included a combination of macrosystem (policy) and exosystem (built environment) solutions, including data-driven focalization to identify areas needing maintenance, the co-design of pedestrian infrastructure with people with disabilities to ensure accessibility and responsiveness to diverse needs, and the construction of bicycle lanes.

To address public safety, they proposed exosystem (built environment) interventions such as the revitalization of abandoned lots, construction of outdoor gyms, surveillance cameras and the expansion of the street lighting network, as well as macrosystem (policy) recommendations, such as a more equitable geographic distribution of police officers and the creation of an intersectoral civic committee with government officials, commercial establishments, universities, community leaders, residents, and transient populations to address high-risk areas.

Citizen scientists also proposed solutions to other themes considered a priority. To address the inappropriate use of public spaces, for instance, they suggested exosystem (built environment) improvements, such as the construction and improved accessibility of public restrooms, as well as strategies to improve the mesosystem (social environment), like educational campaigns to promote responsible use of public spaces and the collaboration between street vendors and commercial establishments to achieve consensus on the governance of public space. As a first step towards cooperation, they proposed a street vendor census to better understand the location and needs of this population.

To address waste in public spaces, they suggested co-designing waste disposal furniture and using tactical urbanism to enhance the visual appeal of areas with high waste accumulation. Moreover, they suggested mesosystem (social environment) strategies such as fines for improper waste disposal, neighborhood awards, and educational campaigns on recycling and responsible waste practices for both professional recyclers and the broader community.

Additional exosystem (built environment) proposals included metro, bicycle, and motorcycle infrastructure to reduce traffic congestion, as well as housing complexes on abandoned lots to support the unsheltered population. Other macrosystem (policy) strategies included subsidizing historically significant housing to mitigate gentrification, the development of a council to monitor compliance with environmental regulations, zoning areas for nightclubs to control noise, new bus and Bus Rapid Transit routes to address deficiencies in public transport operations, and *Pico y Placa* policy (restriction of certain license plates during peak hours) adjustments to ease traffic.

#### Activate: knowledge mobilization of 7th Street liveability facilitators and barriers

A total of 17 citizen scientists and 24 policymakers attended the *Activate step* community meeting. Policymakers represented various district entities, including Bogotá’s District Secretariats for Culture (*n* = 9), Planning (*n* = 4), Mobility (*n* = 3), Social Integration (*n* = 2), Environment (*n* = 1), Health (*n* = 1), Safety (*n* = 1), the Administrative Department of Defense of Public Space (*n* = 2) and TransMilenio (*n* = 1).

At the beginning of the meeting, citizen scientists presented key barriers to urban liveability along with their proposed solutions. Prioritized issues included those identified during the *Discuss Step*, such as the inappropriate use of public space, poor quality or lack of pedestrian infrastructure, and women’s perceived lack of safety in public spaces, as well as broader urban challenges like insufficient institutional response to housing barriers and vulnerable populations’ rights, and deficiencies in public transport operations.

Policymakers addressed the concerns of citizen scientists through three main approaches (Table 4). First, they highlighted ongoing strategies that could benefit from the results of citizen scientists. For example, the District Secretariat of Culture referenced the intersectoral strategy *El Centro Vive* to emphasize the value of citizen scientists’ data in guiding areas that need tactical urbanism interventions and educational campaigns to revitalize public spaces. Academic moderators noted the need to disseminate information about this strategy to foster collaboration among city entities, commercial establishments, and residents. Similarly, the District Secretariat for Mobility mentioned *Local Mobility Centers* as places where citizen scientists could bring their insights to help identify priority areas for improvements in pedestrian infrastructure. More broadly, policymakers acknowledged the value of these contributions in validating current strategies and informing diagnostics for future projects. As one policymaker noted:

The information provided by this research helps consolidate the Secretariat’s diagnosis of the eastern corridor, which is part of our analysis for major infrastructure projects, such as the first metro line. These interventions, including the closure of Caracas stations, will alter urban dynamics. These results will help us refine the traffic management plans for these interventions. (Secretariat for Mobility policymaker)

Second, policymakers leveraged existing initiatives to emphasize the shared responsibility between government and community actors in addressing urban challenges. For instance, the District Secretariat for Planning referenced the *Local Planning Units* within the 2023–2035 Land Use Master Plan as a tool to decentralize planning and allocate budgets locally, encouraging residents to engage in participatory budgeting processes. Moreover, the Secretariat for Safety, Mobility, and TransMilenio highlighted their educational campaigns in public spaces and institutions to combat gender-based violence, noting that cultural change in gender-based violence requires sustained community collaboration. The Secretariat for Social Integration detailed programs that provide shelter, employment, hygiene products, and civic education to unsheltered individuals, while urging residents to cultivate empathy and share responsibility for maintaining public spaces. TransMilenio also promoted its mobile app, which allows users to track buses and report drivers who skip stops, encouraging residents to use the platform to help improve service and reduce fare evasion.

Third, policymakers referenced broader policy frameworks, such as the Sustainable Mobility Plan 2023–2035 and the Disability Policy 2023–2034, without specifically addressing concerns raised by citizen scientists. They acknowledged that citywide interventions are not feasible in the short term but emphasized steady progress toward long-term goals. In addition, they highlighted other service delivery mechanisms, like the noise control operations triggered by citizen alerts, noting that these often face delays due to the high volume of requests.

At the conclusion of the meeting, citizen scientists reflected on the value of the *Our Voice* process. They noted that it enhanced their awareness of both the built and social environments and provided a platform for their voices to be heard by district entities. They also recognized academia as a mediator and contributor to the design of public policies:

This was a very valuable exercise. It helped us see academia not as distant, but as a bridge to understanding public policy. It showed us that beyond statistics, it’s valid for each person to contribute information from their perspective (Secretariat for Culture policymaker).

#### Change: advocating for improvements in 7th street urban environment

As part of the policy engagement component of our advocacy strategy, we held separate meetings with representatives from the Administrative Department of Defense of Public Space (*n* = 3, date: April 11th, 2024), the District Secretariats of Culture, Habitat, Economic Development, and Education, the District Institutes of Tourism, Art, and Cultural Heritage (*n* = 17, date: April 26th, 2024), the District Secretariat of Mobility (*n* = 7, date: August 27th, 2024 & November 5th, 2024) and the Urban Development Institute (*n* = 8; date: March 17th, 2025). During these engagements, policymakers acknowledged the relevance of the data collected and initially analyzed by citizen scientists. They emphasized its potential to enhance both current and future urban interventions by enriching statistical evidence with local experiential knowledge. The data were also recognized as a valuable baseline for future planning and evaluation.

As part of the public communication component of our advocacy strategy, we developed four podcasts and six Instagram reels. As of October 2025, each of the four podcasts was played between 101 and 508 times by listeners of the 070 independent media outlet, and each of the six Instagram reels reached between 3,700 and 7,900 accounts on Instagram. Audience responses included a variety of reflections, such as:

Undoubtedly, it is essential to amplify the voices of people with disabilities, as only then can we truly understand the importance and urgency of adapting the city to ensure universal accessibility. It is a call to authorities, those involved in urban planning and construction, and even to us who inhabit and use public space. (Instagram user)

This testimony deeply resonated with me. I lived in Chapinero for several years until my dog came into my life, and that’s when I realized how grey, dirty, unfriendly, and barely breathable 7th Street really was. Add to that the perception of unsafety that we’ve unfortunately come to normalize, and the lack of green spaces in this highly privileged area. The solution: I moved to another part of the city (…). We felt the difference from the very first day. I miss Chapinero, but my dog probably doesn’t. Do we all fit in our city? Are we aware of how our presence contributes to the liveability of a place? And if we can’t move to another place, then what? (Instagram user)

As a direct outcome of the advocacy strategy, the research team was invited by the Urban Development Institute in June 2025 to participate in a working group to redesign a segment of the Green Corridor project.

## Discussion

This study provides a baseline evaluation of liveability conditions along 7th Street before the construction of the Green Corridor, combining a representative household survey and the *Our Voice* citizen science method to (i) identify perceived social environment and built environment facilitators and barriers to liveability on 7th Street and (ii) document potential solutions to address the identified barriers. The survey established the population-level context before the project’s implementation, documenting who lives along the corridor and the expected effects of the intervention, while the *Our Voice* method captured the lived experiences and community-generated solutions. Integrating these two components revealed that the conditions residents most value and fear losing are those that citizen scientists identified as the defining features of liveability along 7th Street: green and pedestrian infrastructure, public space quality, and safety. Proposed solutions by citizen scientists spanned the exosystem (built environment), mesosystem (social environment), and macrosystem (policy), including infrastructure upgrades, educational campaigns, and evidence-based policymaking. Policymakers responded by either referencing strategies that could be strengthened with *Our Voice* data, promoting shared responsibility, or aligning community concerns with broader urban frameworks. While rooted in the local context of Bogotá’s 7th Street, this study offers generalizable insights into the value of multi-method, participatory approaches for informing urban transformation within broader city agendas. Baseline data are essential for the design and evaluation of future effectiveness interventions.

Our results support the idea that liveability is shaped by exosystem (built environment), mesosystem (social environment), and macrosystem (policy) across different urban domains. Facilitators identified by citizen scientists, such as access to essential services and green infrastructure, are consistently linked to improved health outcomes in high-income settings and Latin America, including a reduced risk of chronic diseases and increased mental health [39–42]. Barriers such as safety concerns and sidewalk quality are also consistent with findings from high-income countries, particularly regarding physical activity, wellbeing, and fall risk [40, 43–45]. These findings directly support the current design priorities of 7th Street Green Corridor, where the transformation of green areas and pedestrian infrastructure is central. This is particularly relevant given the resistance that this intervention has faced from some communities [46].

Gendered differences also emerged, reinforcing the distinct experiences that population groups face in urban environments. Although we did not elaborate on a full disaggregated analysis by gender, the women in our study emphasized the need for safer and more accessible cities. This is consistent with global research on gendered experiences of urban environments, where women tend to perceive public and transport spaces as less secure due to poor-quality infrastructure and the high prevalence of sexual harassment [47–49]. These findings reflect not only individual experiences but also social and spatial manifestations of the broader systemic issue of gendered power dynamics and violence [50]. Urban planning and design have historically failed to include a gender-sensitive perspective and often have systematically excluded women from decision-making processes, thereby perpetuating structural gender inequalities that limit women’s autonomy, mobility, and full participation in public life [51, 52]. These findings underscore the importance of integrating safety measures into the design of the Green Corridor or other interventions, recognizing that physical transformation alone is insufficient for the intervention to be truly equitable and ensure that all residents, particularly women and vulnerable groups, can safely occupy and benefit from public space. The range of solutions proposed by citizen scientists, spanning the exosystem (built environment), mesosystem (social environment), and macrosystem (policy), further reflects this understanding, as promoting liveability requires simultaneous action across multiple dimensions, not only physical ones.

Our results show that residents’ expectations largely align with the conceptual aims of the Green Corridor, yet this alignment does not guarantee full community support for the intervention. As seen in other cities like Barcelona’s Poblenou Superblock (Spain) [53], residents may raise concerns about governance and unintended consequences, such as gentrification. In our study, respondents expressed both optimism and apprehension about the Green Corridor’s impact, underscoring the need for participatory planning processes that anticipate and mitigate potential negative effects. The *Our Voice* findings further suggest that residents consider meaningful community involvement essential to foster collective ownership and long-term sustainability, a dimension that the survey could not capture.

Our findings also underscore the shared understanding between residents and policymakers that incorporating lived experience into the design of urban interventions is a promising step toward participatory planning. However, they also reveal a tendency among policymakers to rely on existing programs when responding to residents’ concerns, rather than co-creating new solutions. This mirrors challenges documented across Latin American cities, where translating citizen input into action is hindered by resource constraints, limited authority, and institutional inertia [54–57]. To bridge this gap, our team developed an advocacy strategy that led to an invitation to participate in the redesign of the Green Corridor, illustrating the potential of citizen science to influence policy when supported by strategic engagement. More broadly, these findings suggest the need for formal systems to capture community perceptions during the planning and design stages of urban interventions. Establishing systematic channels for community engagement before implementation could help ensure that interventions like the Green Corridor are designed with, not just for, the communities they aim to serve.

Citizen scientists’ participation in generating solutions, especially among previously uninvolved residents, increased their awareness of their surroundings and allowed them to formulate concrete responses to shared urban challenges. Findings from other *Our Voice* studies align with ours, demonstrating that community engagement fosters greater civic awareness and encourages solution-oriented thinking [34, 35]. Given that 31 additional Green Corridors are planned across Bogotá, this experience offers a replicable model for grounding future urban transformations in the lived experiences and solutions of the communities they will affect.

The following limitations should be considered when interpreting our findings. First, while our sample in the community-engaged project captured diverse perspectives, it may not fully represent all social groups in Bogotá, such as youth, people with severe disabilities, or those with extreme vulnerability. To address this limitation, we complemented citizen scientists’ perspectives with a representative survey of residents living along 7th Street. Second, while the Our Voice app effectively documented visible barriers, it may have missed less visible issues like gender-based violence or social dynamics within the territory. To address this, we accompanied citizen scientists during their walk audits, asking them questions to identify additional, less visible barriers. Third, the three *Discuss* step community meetings were organized by residential address rather than commuting patterns, which may have influenced the nature of the discussions: the former sheds light on place-based experiences while the latter reveals insights shaped by mobility. In our study, only 7.1% of the photo-narratives were captured outside participants’ home segments, minimizing this risk. Fourth, attrition among citizen scientists was observed at each stage of the Our Voice process. Specifically, 31 participants disengaged between the *Discover* and *Discuss* steps due to scheduling conflicts, and another 18 dropped out between the *Discuss* and *Activate* steps after an eight-month gap between meetings caused by mayoral elections. This may have limited the diversity of perspectives during the *Activate* step. Future initiatives should consider political and social timelines to ensure continuity and support effective policy advocacy.

## Conclusion

This study underscores the importance of grounding climate mitigation and adaptation urban transformations in baseline assessments that capture both the breadth and depth of residents’ liveability experiences. By combining a representative household survey with the *Our Voice* citizen science method, we documented the social and built environment conditions that facilitate and hinder liveability along 7th Street before the Green Corridor’s construction. The survey established that built environment factors are widely valued as facilitators across the population, while social environment factors emerge as persistent barriers, a pattern the *Our Voice* method deepened by revealing the specific conditions that underpin these perceptions. We also documented potential actionable solutions that span infrastructure, educational campaigns, and evidence-based policymaking. The participatory process deepened citizen scientists’ awareness of their urban surroundings and enabled meaningful engagement with policymakers to address shared urban concerns. As cities pursue climate goals, these findings offer a foundation for understanding and evaluating the effects of a Green Corridor on residents’ everyday liveability as the intervention unfolds.

## Supplementary Information

Additional File 1: Questionnaire from the cross-sectional household survey assessing whether the Green Corridor intervention is likely to improve or reduce liveability along 7th Street.

Additional File 2: Matrices of liveability facilitators and barriers identified by citizen scientists during the Discover step.

## Data Availability

Data generated during the current study are not publicly available due to privacy reasons. De-identified photovoice and survey data may be available from the corresponding author on reasonable request.
