# Supplementary material for: Community voices to understand and promote liveability in the Green Corridor urban transformation project in Bogotá, Colombia
Source: BMC Public Health. 2026 May 16;26:2078. doi: 10.1186/s12889-026-27578-9 (PMC13348599; doi:10.1186/s12889-026-27578-9)
Supplement: Supplementary file 1 — Additional File 1: Questionnaire from the cross-sectional household survey assessing whether the Green Corridor intervention is likely to improve or reduce liveability along 7th Street. [file 12889_2026_27578_MOESM1_ESM.docx]

**Additional File 1: Questionnaire from the cross-sectional household survey assessing whether the Green Corridor intervention is likely to improve or reduce liveability along 7th Street**

| 1. Which specific interventions proposed by the Green Corridor project are you familiar with?  (INTERVIEWER: SPONTANEOUS RESPONSE; MULTIPLE ANSWERS).  Prioritizes or increases space for pedestrians… 1  Prioritizes or increases space for bicycles 2  Improves public space in general 3  Improves public transport 4  Improves road safety 5  More green areas and trees 6  Improves mobility and encourages sustainable mobility 7  Improves urban furniture (benches, bus stops, poles, trash cans) 8  Improves the appearance and aesthetics of 7^th^ Street 9  Improves safety regarding crimes or offenses 10  Reduces environmental pollution 11  Promotes commerce and economic development 12  Promote spaces for exercise, sports, and recreation 13  Facilitates social, community, cultural activities and improves social infrastructure 14  None 15  Other 88  Don’t know/No answer 99 |  | 2. Using a scale from 1 to 5, where 5 means 'Strongly agree' and 1 means 'Strongly disagree', how much do you agree with the Green Corridor project planned on 7^th^ Street?  (INTERVIEWER: SHOW CARD 2, ONE ANSWER)  Strongly disagree 1  2  3  4  Strongly Agree 5  Don’t know/No answer (I: DO NOT READ) 99 |
| --- | --- | --- |
| 3. What are the three aspects you like most about the Green Corridor project? Tell me the option you like most.  (INTERVIEWER: SPONTANEOUS RESPONSE, ONE ANSWER)  Prioritizes or increases space for pedestrians… 1  Prioritizes or increases space for bicycles 2  Improves public space in general 3  Improves public transport 4  Improves road safety 5  More green areas and trees 6  Improves mobility and encourages sustainable mobility 7  Improves urban furniture (benches, bus stops, poles, trash cans) 8  Improves the appearance and aesthetics of 7th Street 9  Improves safety regarding crimes or offenses 10  Reduces environmental pollution 11  Promotes commerce and economic development 12  Promote spaces for exercise, sports, and recreation 13  Facilitates social, community, cultural activities and improves social infrastructure 14  None 15  Other 88  Don’t know/No answer 99 |  | 4. What are the three aspects you like most about the Green Corridor project? Tell me the second option you like most.  (INTERVIEWER: SPONTANEOUS RESPONSE, ONE ANSWER, DO NOT REPEAT OPTIONS FROM THE PREVIOUS QUESTION)  Prioritizes or increases space for pedestrians… 1  Prioritizes or increases space for bicycles 2  Improves public space in general 3  Improves public transport 4  Improves road safety 5  More green areas and trees 6  Improves mobility and encourages sustainable mobility 7  Improves urban furniture (benches, bus stops, poles, trash cans) 8  Improves the appearance and aesthetics of 7th Street 9  Improves safety regarding crimes or offenses 10  Reduces environmental pollution 11  Promotes commerce and economic development 12  Promote spaces for exercise, sports, and recreation 13  Facilitates social, community, cultural activities and improves social infrastructure 14  None 15  Other 88  Don’t know/No answer 99 |
| 5. What are the three aspects you like most about the Green Corridor project? Tell me the third option you like most.  (INTERVIEWER: SPONTANEOUS RESPONSE, ONE ANSWER, DO NOT REPEAT OPTIONS FROM THE PREVIOUS QUESTIONS)  Prioritizes or increases space for pedestrians… 1  Prioritizes or increases space for bicycles 2  Improves public space in general 3  Improves public transport 4  Improves road safety 5  More green areas and trees 6  Improves mobility and encourages sustainable mobility 7  Improves urban furniture (benches, bus stops, poles, trash cans) 8  Improves the appearance and aesthetics of 7th Street 9  Improves safety regarding crimes or offenses 10  Reduces environmental pollution 11  Promotes commerce and economic development 12  Promote spaces for exercise, sports, and recreation 13  Facilitates social, community, cultural activities and improves social infrastructure 14  None 15  Other 88  Don’t know/No answer 99 |  | 6. What are the three aspects you like least about the Green Corridor project? Tell me the option you like least.  (INTERVIEWER: SPONTANEOUS RESPONSE, ONE ANSWER)  Negatively affects space for pedestrians 1  Negatively affects space for bicycles 2  Worsens public space 3  Worsens public transport 4  Prioritizes TransMilenio, I prefer other alternatives 5  Affects road safety 6  Damages green areas 7  Reduces number of lanes for cars and motorcycles 8  Negatively affects appearance and aesthetics of 7^th^ Street, I don't like the design 9  More insecurity, thefts, crimes 10  Hinders access to properties and parking for private vehicles 11  Worsens air pollution 12  Worsens noise pollution 13  Negatively affects commerce and economic development 14  Hinders car use 15  Other 88  Don’t know/No answer 99 |
| 7. What are the three aspects you like least about the Green Corridor project? Tell me the second option you like least.  (INTERVIEWER: SPONTANEOUS RESPONSE, ONE ANSWER, DO NOT REPEAT OPTIONS FROM THE PREVIOUS QUESTION)  Negatively affects space for pedestrians 1  Negatively affects space for bicycles 2  Worsens public space 3  Worsens public transport 4  Prioritizes TransMilenio, I prefer other alternatives 5  Affects road safety 6  Damages green areas 7  Reduces number of lanes for cars and motorcycles 8  Negatively affects appearance and aesthetics of 7^th^ Street, I don't like the design 9  More insecurity, thefts, crimes 10  Hinders access to properties and parking for private vehicles 11  Worsens air pollution 12  Worsens noise pollution 13  Negatively affects commerce and economic development 14  Hinders car use 15  Other 88  Don’t know/No answer 99 |  | 8. What are the three aspects you like least about the Green Corridor project? Tell me the third option you like least.  (INTERVIEWER: SPONTANEOUS RESPONSE, ONE ANSWER, DO NOT REPEAT OPTIONS FROM THE PREVIOUS QUESTIONS)  Negatively affects space for pedestrians 1  Negatively affects space for bicycles 2  Worsens public space 3  Worsens public transport 4  Prioritizes TransMilenio, I prefer other alternatives 5  Affects road safety 6  Damages green areas 7  Reduces number of lanes for cars and motorcycles 8  Negatively affects appearance and aesthetics of 7^th^ Street, I don't like the design 9  More insecurity, thefts, crimes 10  Hinders access to properties and parking for private vehicles 11  Worsens air pollution 12  Worsens noise pollution 13  Negatively affects commerce and economic development 14  Hinders car use 15  Other 88  Don’t know/No answer 99 |
| 9. Regarding your gender, you identify as:  (INTERVIEWER: ONE ANSWER)  Woman 1  Man 2  Other 88  Don’t know/Not applicable 99 |  | 10. According to your culture, people, or physical traits, you identify as:  (INTERVIEWER: ONE ANSWER)  Indigenous 1  Black / Afro-descendant / Mulato 2  Gypsy or Rrom 3  Raizal 4  Palenquero 5  White 6  Mestizo 7  Other 8  No ethnic group 9 |
| 11. What is your marital status?  (INTERVIEWER: ONE ANSWER)  Single 1  Widowed 2  Married 3  Common-law partner 4  Divorced or Separated 5 |  | 12. Thinking about your relationship to the head of the household, you are:  (INTERVIEWER: SPONTANEOUS RESPONSE, ONE ANSWER)  Head of household 1  Spouse or partner of the head of household 2  Child of the head of household 3  Stepchild of the head of household 4  Grandchild of the head of household 5  Parent of the head of household… 6  Sibling or stepsibling of the head of household 7  Parent-in-law of the head of household 8  Son-in-law, daughter-in-law of the head of household 9  Other relative of the head of household 10  Other non-relative 11 |
| 13. What is the highest educational level you have reached (even if not completed)?  (INTERVIEWER: SPONTANEOUS RESPONSE, ONE ANSWER)  None 1  Preschool 2  Basic / Incomplete Primary 3  Basic / Complete Primary 4  Basic / Incomplete High School 5  Basic / Complete High School 6  Higher education, incomplete technical or technological 7  Higher education, completed technical or technological 8  Incomplete undergraduate / University 9  Completed undergraduate / University 10  Postgraduate / Specialization / Master's / Doctorate 11 |  | 14. What is the last year completed at this educational level?  (INTERVIEWER: SPONTANEOUS RESPONSE) |
| 15. What activity occupied most of your time last week?  (INTERVIEWER: ONE ANSWER)  Working in a company or government 1  Working independently or self-employed 2  Did not work, but had a job 3  Looking for work 4  Studying 5  Domestic employee 6  Employer 7  Household chores or unpaid care work 8  Unpaid family worker 9  Unpaid worker in businesses of other households 10  Day laborer 11  Retired 12  Not working due to disability or prolonged illness 13  Other 88 |  | 16. Could you tell me in which of the following ranges your household's monthly income falls, considering all sources of income during the last month?  (INTERVIEWER: SHOW CARD 12, ONE ANSWER)  0 to 1 Minimum Wage (COP $0 - $1,160,000) 1  More than 1 to 2 Minimum Wages (COP $1,160,001 - $2,320,000) 2  More than 2 to 4 Minimum Wages (COP $2,320,001 - $4,640,000) 3  More than 4 to 16 Minimum Wages (COP $4,640,001 - $18,560,000) 4  More than 16 Minimum Wages (More than COP $18,560,000) 5  Domestic employee 6  Employer 7  Household chores or unpaid care work 8  Unpaid family worker 9  Unpaid worker in businesses of other households 10  Day laborer 11  Retired 12  Not working due to disability or prolonged illness 13  Don’t know/No answer (I: DO NOT READ) 99 |
