# Supplementary material for: Community voices to understand and promote liveability in the Green Corridor urban transformation project in Bogotá, Colombia
Source: BMC Public Health. 2026 May 16;26:2078. doi: 10.1186/s12889-026-27578-9 (PMC13348599; doi:10.1186/s12889-026-27578-9)
Supplement: Supplementary file 2 — Additional File 2: Matrices of liveability facilitators and barriers identified by citizen scientists during the Discover step. [file 12889_2026_27578_MOESM2_ESM.docx]

**Additional File 2: Matrices of liveability facilitators and barriers identified by citizen scientists during the *Discover step***

**Table s1.** Matrix of liveability facilitators identified by citizen scientists during the *Discover step*, categorized by liveability domains and socioecological levels across 7^th^ Street segments.

| **Socioecological level** | **Themes** | **S1** | **S2** | **S3** |
| --- | --- | --- | --- | --- |
| **Liveability environment domain** | | | | |
| Exosystem: Built environment | Presence of green areas and urban trees | X | X | X |
| Mesosystem: Social environment | Environmentally sustainable citizen practices | X | X | X |
| Macrosystem: Policy | Institutional response to the recovery of green areas |  |  | X |
| **Liveability infrastructure domain** | | | | |
| Exosystem: Built environment | Access to cultural and community gathering spaces | X | X | X |
|  | Appropriate organization of informal vendors to trade in public spaces | X |  |  |
|  | Beautification and aesthetic quality of public spaces | X | X | X |
|  | Good quality of urban furniture | X | X |  |
|  | Good quality or presence of bicycle infrastructure | X | X | X |
|  | Good quality of vehicular infrastructure |  | X | X |
|  | Good quality of pedestrian infrastructure | X | X | X |
|  | Pedestrianization of public vehicular infrastructure | X |  |  |
|  | Presence and proximity to essential services |  | X | X |
|  | Presence of spaces and services for sports and physical activity | X | X | X |
| Mesosystem: Social environment | Adequate waste disposal practices |  |  | X |
|  | Active use of public space |  | X |  |
|  | Maintenance of urban furniture by citizens |  | X |  |
|  | Positive experience of cross-cultural interaction | X | X |  |
|  | Recognition of the historical and cultural relevance of the area | X | X |  |
| Macrosystem: Policy | Conservation, recovery, and revitalization of public spaces | X | X | X |
|  | Implementation of sustainable mobility infrastructure | X | X | X |
|  | Institutional control of tourism practices | X |  |  |
|  | **Liveability mobility domain** |  |  |  |
| Macrosystem: Built environment | Ease of access to public transport | X | X | X |
|  | Efficiency in public transit operating hours |  | X | X |
|  | Good public transport coverage | X | X | X |
|  | Multiplicity of transport modes | X | X | X |
|  | Reduced travel time due to the presence of exclusive public transport lanes | X |  |  |
| Mesosystem: Social environment | Traffic law compliance due to surveillance cameras |  | X |  |
| **Liveability housing and employment domain** | | | | |
| Macrosystem: Built environment | Adequate access to public services |  |  | X |
|  | Diverse sources of employment |  | X | X |
|  | Presence of residential areas |  |  | X |
|  | Proximity between home and work |  | X |  |
| Mesosystem: Social environment | Community action to respond to gentrification | X |  |  |
|  | Income derived from commerce in public space | X | X |  |
|  | Positive perception of gentrification | X |  |  |
| **Liveability safety domain** | | | | |
| Mesosystem: Social environment | Safety perception in public space | X | X | X |
| Macrosystem: Policy | Safety perception due to the effective management of public and private safety entities | X | X | X |

Table s1 presents liveability facilitators identified by citizen scientists during the *Discover* step, categorized by liveability domains and socioecological levels across 7^th^ Street segments. Themes are organized under three socioecological levels (mesosystem: social environment, exosystem: built environment, macrosystem: policy levels) grouped into five liveability domains (environment, infrastructure, mobility, housing and employment, and safety). The presence of each facilitator in one or more segments (S1, S2, S3) is indicated with an “X”.

**Table s2.** Matrix of liveability barriers identified by citizen scientists during the *Discover step*, categorized by liveability domains and socioecological levels across 7^th^ Street segments.

| **Socioecological level** | | **Themes** | **S1** | **S2** | **S3** |
| --- | --- | --- | --- | --- | --- |
|  | **Liveability environment domain** | | | | |
| Exosystem: Built environment | | Lack of tree cover and green areas | X | X | X |
|  |  | Landslide risk |  |  | X |
|  |  | Poor air quality | X | X | X |
|  |  | Transformation of the natural environment due to urbanization |  | X | X |
| Mesosystem: Social environment | | Pollution due to low environmental education | X | X | X |
| Macrosystem: Policy | | Insufficient institutional response to environmental barriers |  |  | X |
|  |  | Insufficient institutional response to promote environmental sustainability |  | X |  |
|  |  | Lack of awareness of communication channels with entities to address environmental barriers |  |  | X |
| **Liveability infrastructure domain** | | | | | |
| Exosystem: Built environment | | Absence and lack of proximity to essential services |  | X | X |
|  |  | Bad odors in public spaces | X | X | X |
|  |  | Deficient sanitation infrastructure | X | X | X |
|  |  | Deterioration and loss of cultural heritage | X |  |  |
|  |  | Lack of access to cultural and community gathering spaces |  | X | X |
|  |  | Lack of spaces for sports and physical activity | X |  |  |
|  |  | Poor quality or lack of cycling infrastructure | X | X | X |
|  |  | Poor quality or lack of parks |  |  | X |
|  |  | Poor quality or lack of pedestrian infrastructure | X | X | X |
|  |  | Poor quality or lack of urban furniture | X | X | X |
|  |  | Poor quality or lack of vehicular infrastructure | X | X | X |
|  |  | Presence of stray dogs |  |  | X |
|  |  | Presence of waste in public space | X | X | X |
|  |  | Resistance to the transformation of 7^th^ Street |  | X | X |
|  |  | Visual pollution from advertising |  |  | X |
| Mesosystem: Social environment | | Consumption and/or sale of psychoactive substances in public spaces | X |  | X |
|  |  | Crowding of people in public spaces | X |  |  |
|  |  | Disorganized presence of informal vendors | X | X | X |
|  |  | Inadequate handling of food offered in public spaces | X |  |  |
|  |  | Inadequate waste disposal practices | X | X | X |
|  |  | Lack of care for public spaces | X | X | X |
|  |  | Lower wellbeing and quality of life due to public disorder during demonstrations | X |  |  |
|  |  | Marginalization and discrimination of social groups in public spaces | X | X |  |
|  |  | Noise pollution | X | X | X |
| Macrosystem: Policy | | Barriers to citizen participation in public and social infrastructure issues | X |  |  |
|  |  | Insufficient institutional response to the organization of informal vendors |  | X |  |
|  |  | Insufficient institutional response to public space barriers | X | X | X |
|  |  | Insufficient institutional response to vulnerable populations’ rights | X |  |  |
| **Liveability mobility domain** | | | | | |
| Exosystem: Built environment | | Barriers to access to public transport | X | X | X |
|  |  | Cyclists’ risk of incidents due to a lack of or poor quality of bicycle infrastructure | X | X | X |
|  |  | Deficiencies in the scheduled operation of public transport | X | X | X |
|  |  | High cost of public transport |  |  | X |
|  |  | Lack of public transport coverage | X | X | X |
|  |  | Long travel times in public transport |  | X | X |
|  |  | Mobility obstruction due to construction | X | X | X |
|  |  | Motorized vehicles’ risk of incidents due to the poor quality of vehicular infrastructure |  | X | X |
|  |  | Pedestrians’ risk of incidents due to a lack of or poor quality of pedestrian infrastructure | X | X | X |
|  |  | Traffic congestion derived from deficient vehicular infrastructure |  | X | X |
|  |  | Traffic congestion |  | X | X |
| Mesosystem: Social | | Cyclists’ risk of incidents due to the inappropriate behavior of road users |  | X | X |
| environment | | Fluctuations in occupancy and overcrowding of passengers in public transport | X | X | X |
|  |  | Inappropriate behavior of road users | X | X |  |
|  |  | Mobility obstruction due to the invasion of roads and sidewalks | X | X | X |
|  |  | Pedestrians’ risk of incidents due to the inappropriate behavior of road users | X | X | X |
| Macrosystem: Policy | | Insufficient institutional response to mobility barriers | X |  | X |
| **Liveability housing and employment domain** | | | | | |
| Exosystem: Built environment | | Long distance between housing and employment |  | X | X |
|  |  | Negative impacts caused by new constructions | X |  | X |
|  |  | Presence of informal settlements |  | X | X |
|  |  | Properties in a state of abandonment | X | X | X |
| Mesosystem: Social environment | | Difficulty accessing employment |  | X | X |
|  |  | Gentrification and high cost of living | X | X | X |
|  |  | Violation of the rights of children and adolescents | X |  |  |
| Macrosystem: Policy | | Insufficient institutional response regarding bar regulation |  | X |  |
|  |  | Insufficient institutional response to guaranteeing labor rights for vulnerable population groups | X |  |  |
|  |  | Insufficient institutional response to housing barriers |  |  | X |
| **Liveability safety domain** | | | | | |
| Exosystem: Built environment | | Lack of safety perception derived from the built environment in public spaces | X | X | X |
| Mesosytem: Social environment | | Lack of safety perception derived from the social environment in public spaces | X | X | X |
| Macrosystem: Policy | | Insufficient institutional response to safety barriers | X | X | X |

Table s2 presents liveability barriers identified by citizen scientists during the *Discover* step, categorized by liveability domains and socioecological levels across 7^th^ Street segments. Themes are organized under three socioecological levels (mesosystem: social environment, exosystem: built environment, macrosystem: policy levels) grouped into five liveability domains (environment, infrastructure, mobility, housing and employment, and safety). The presence of each barrier in one or more segments (S1, S2, S3) is indicated with an “X”.
